# Australasian Malignant PLeural Effusion (AMPLE)-3 trial: study protocol for a multi-centre randomised study comparing indwelling pleural catheter (±talc pleurodesis) versus video-assisted thoracoscopic surgery for management of malignant pleural effusion

**DOI:** 10.1186/s13063-022-06405-7

**Published:** 2022-06-27

**Authors:** Deirdre B. Fitzgerald, Calvin Sidhu, Charley Budgeon, Ai Ling Tan, Catherine A. Read, Benjamin C. H. Kwan, Nicola Ann Smith, Edward T. Fysh, Sanjeevan Muruganandan, Tajalli Saghaie, Ranjan Shrestha, Arash Badiei, Phan Nguyen, Andrew Burke, John Goddard, Morgan Windsor, Julie McDonald, Gavin Wright, Kasia Czarnecka, Parthipan Sivakumar, Kazuhiro Yasufuku, David J. Feller-Kopman, Nick A. Maskell, Kevin Murray, Y. C. Gary Lee

**Affiliations:** 1 Respiratory Medicine, Sir Charles Gairdner Hospital, Nedlands, WA Australia; 2 Medical School, Faculty of Health & Medical Sciences, University of Western Australia, Perth, WA Australia; 3 Pleural Medicine Unit, Institute for Respiratory Health, Perth, WA Australia; 4 School of Medical and Health Sciences, Edith Cowan University, Joondalup, WA Australia; 5 School of Population and Global Health, University of Western Australia, Perth, WA Australia; 6 Department of Respiratory and Sleep Medicine, The Sutherland Hospital, Sydney, NSW Australia; 7 University of New South Wales, Sydney, NSW Australia; 8 Respiratory Department, Wellington Regional Hospital, Wellington, New Zealand; 9 Respiratory Medicine, St John of God Hospital Midland, Midland, WA Australia; 10 Respiratory Medicine, Northern Health, Epping, VIC Australia; 11 Respiratory Medicine, Concord Repatriation General Hospital, Concord West, NSW Australia; 12 Faculty of Medicine and Health Sciences, Macquarie University, Sydney, NSW Australia; 13 Respiratory Medicine, Fiona Stanley Hospital, Murdoch, WA Australia; 14 Thoracic Medicine, Royal Adelaide Hospital, Adelaide, SA Australia; 15 Adelaide Medical School, Faculty of Health and Medical Science, University of Adelaide, Adelaide, SA Australia; 16 Thoracic Medicine, The Prince Charles Hospital, Brisbane, QLD Australia; 17 School of Medicine, The University of Queensland School of Medicine, Brisbane, QLD Australia; 18 Respiratory Department, Sunshine Coast University Hospital, Birtinya, QLD Australia; 19 Griffith University, Brisbane, QLD Australia; 20 Thoracic Surgery, The Prince Charles Hospital, Brisbane, QLD Australia; 21 Respiratory and Sleep Medicine Department, St Vincent’s Hospital, Melbourne, VIC Australia; 22 Department of Cardiothoracic Surgery & University of Melbourne Department of Surgery, St Vincent’s Hospital, Melbourne, VIC Australia; 23 Division of Thoracic Surgery, Toronto General Hospital University Health Network, University of Toronto, Toronto, Ontario Canada; 24 Guy’s and St Thomas’ NHS Foundation Trust, London, UK; 25 Department of Medicine, Johns Hopkins University, Baltimore, MD USA; 26 Academic Respiratory Unit, Bristol Medical School, University of Bristol, Bristol, UK

**Keywords:** Pleural effusion, Indwelling pleural catheter, Malignant, Video-assisted thoracoscopic surgery, Randomised controlled trial

## Abstract

**Introduction:**

Malignant pleural effusions (MPEs) are common. MPE causes significant breathlessness and impairs quality of life. Indwelling pleural catheters (IPC) allow ambulatory drainage and reduce hospital days and re-intervention rates when compared to standard talc slurry pleurodesis. Daily drainage accelerates pleurodesis, and talc instillation via the IPC has been proven feasible and safe. Surgical pleurodesis via video-assisted thoracoscopic surgery (VATS) is considered a one-off intervention for MPE and is often recommended to patients who are fit for surgery. The AMPLE-3 trial is the first randomised trial to compare IPC (±talc pleurodesis) and VATS pleurodesis in those who are fit for surgery.

**Methods and analysis:**

A multi-centre, open-labelled randomised trial of patients with symptomatic MPE, expected survival of ≥ 6 months and good performance status randomised 1:1 to either IPC or VATS pleurodesis. Participant randomisation will be minimised for (i) cancer type (mesothelioma vs non-mesothelioma); (ii) previous pleurodesis (vs not); and (iii) trapped lung, if known (*vs* not). Primary outcome is the need for further ipsilateral pleural interventions over 12 months or until death, if sooner. Secondary outcomes include days in hospital, quality of life (QoL) measures, physical activity levels, safety profile, health economics, adverse events, and survival. The trial will recruit 158 participants who will be followed up for 12 months.

**Ethics and dissemination:**

Sir Charles Gairdner and Osborne Park Health Care Group (HREC) has approved the study (reference: RGS356). Results will be published in peer-reviewed journals and presented at scientific meetings.

**Discussion:**

Both IPC and VATS are commonly used procedures for MPE. The AMPLE-3 trial will provide data to help define the merits and shortcomings of these procedures and inform future clinical care algorithms.

**Trial registration:**

Australia New Zealand Clinical Trial Registry ACTRN12618001013257. Registered on 18 June 2018.

Protocol version: Version 3.00/4.02.19

**Supplementary Information:**

The online version contains supplementary material available at 10.1186/s13063-022-06405-7.

## Introduction

Malignant pleural effusion (MPE) affects over 10,000 Australians and an estimated 5 million people worldwide each year [1]. MPE can complicate most cancers, including 30% of breast and lung cancers and more than 90% of malignant pleural mesotheliomas [2]. The resultant breathlessness is disabling and significantly impairs quality of life. Key goals of management are to relieve breathlessness and enable physical activity while minimising interventions and time spent in hospital, in a cost-effective manner. MPE is associated with a major healthcare burden with annual care costs of over $5 billion in the USA alone [3].

MPE usually indicates incurable cancer with a variable prognosis from days to years; median survival can be as short as four months in lung carcinomas and 12 months in mesothelioma [4–6]. Prognosis depends on multiple factors including performance status and tumour type [4]. Younger, fitter patients, with a prognostically favourable tumour type can be expected to have a significantly longer survival than those with comorbidities and less favourable cancers and, therefore, may warrant significantly different MPE management approaches. A definitive procedure to prevent re-accumulation of effusion post-drainage is typically required to reduce repeated interventions, hospitalisations, and complications. Various treatments with definitive intent of fluid control are available but each has its advantages and limitations.

Surgical pleurodesis is viewed by many clinicians as a one-off intervention with high long-term success rates. Video-assisted thoracoscopic surgery (VATS) has replaced open thoracotomy as the preferred surgical procedure for pleurodesis [7]. If successful, VATS pleurodesis can provide lifelong freedom from the effusion and is the first-line therapy in many institutions worldwide [8]. Reported success rates of 68 to 100% are based mainly on retrospective or single-centre studies [9–12]. One retrospective review of mesothelioma found a re-intervention rate of 32% in the subgroup of patients who underwent VATS pleurodesis at a median of 30 days post-surgery, which was not significantly different from the bedside talc slurry pleurodesis subgroup [13]. Disadvantages of VATS include complications such as fever, pneumonia, and prolonged air leak, occurring in up to 28% of cases [9–12, 14–20]. Post-VATS intercostal neuralgia is also a common long-term complication affecting 25% of patients [21]. VATS pleurodesis requires a median of 5.8 to 10.3 days of hospital stay [12, 15]. The frequent need for general anaesthesia and single lung ventilation also limit the availability of the procedure to patients who are considered fitter and have longer expected survival.

The limitations of surgical pleurodesis have encouraged the development of alternative approaches. Indwelling pleural catheter (IPC) is an ambulatory drainage device for patients with MPE that can be managed on an outpatient basis. A tunnelled catheter is sited and patients (and/or their carers) are educated in home drainage. Multiple prospective studies in the past decade have firmly established IPC as a strong alternative to talc slurry pleurodesis [22, 23]. IPC offers a significant reduction in the need for further interventions for drainage of symptomatic pleural fluid. In prior trials, only 2–6% of IPC-treated patients required further pleural drainages (compared with over 20% in conventional talc pleurodesis) [9, 22, 23]. IPC is also associated with a reduction in hospital days *vs* talc slurry pleurodesis [22–24]. IPC provides effective palliation in the presence of trapped lung, a cohort in whom talc slurry pleurodesis fails due to lack of visceral and parietal pleural apposition [25, 26].

Spontaneous pleurodesis occurs in 40–51% of patients with IPC at a median of 59.0–80.5 days, allowing removal of the IPC [6, 23, 24, 27], especially if the IPC is drained aggressively (e.g., daily) [28, 29]. Talc slurry instillation via IPC has been shown to be safe and feasible with pleurodesis success rates of > 90% in two prospective case series [30, 31]. The feasibility of this combined (IPC + talc) approach was confirmed in a randomised controlled trial (RCT) [32]. Combining IPC with talc pleurodesis and daily drainage may improve outcomes. IPC has its own unique range of complications such as infection (~ 5%) [33, 34], symptomatic loculations (8–13%) [6, 27, 35], and catheter tract metastases (10%) [36]. IPC care can be time consuming and, for patients with better prognoses, may become a burden. IPC also requires ongoing consumables that may result in reduced cost-effectiveness with longer survival [37–39].

Clinical equipoise exists regarding the optimal treatment for MPE, and there is significant dichotomy worldwide. Due to a paucity of high-quality data, decisions are often based on clinician bias and available resources. VATS pleurodesis is conventionally regarded as the optimal approach to provide long-term control of MPE in patients who are fit for surgery. While the role of IPC over bedside talc slurry pleurodesis in the MPE population has been proven, RCTs of IPC (± pleurodesis) to date have investigated patients with advanced cancers, approximately one third of study patients dying within 3 months. Studies of IPC in patients with better performance status and predicted survival, who would also be suitable for VATS pleurodesis, are overdue. Whether IPC combined with talc pleurodesis, and daily drainage would be superior to VATS pleurodesis is unknown. A prospective randomised comparison is required to answer this question.

## Methods

### Study design

The Australian Malignant PLeural Effusion (AMPLE) trial-3 is a multi-centre, open-labelled, randomised (trial entry) study to determine the comparative efficacy of two frequently applied interventions for MPE (VATS or IPC) in improving patient-related clinical outcomes.

### Study setting, participant screening, selection, and recruitment

This trial will be conducted at tertiary centres across Australia, New Zealand, Canada, and the UK. Currently involved institutions are listed in the ethics section. The full list of enrolling sites will be updated regularly on the trial registration website (URL: ACTRN12618001013257). The site investigator or designated research staff will screen patients in both inpatient and outpatient settings. Consecutive eligible patients will be offered trial entry and screening logs will be kept.

Research teams at each site will be contacted regularly by the lead site to discuss hurdles to recruitment. Enrolment and screening logs, including reasons for non-inclusion, will be maintained and de-identified logs will be sent to the lead site monthly.

### Inclusion criteria

Patients with symptomatic MPE that requires definitive intervention, with a predicted survival of more than 6 months and an Eastern Cooperative Oncology Group (ECOG) performance status of 0 to 1 will be included. Patients with a performance status ≥ 2 may be included if it is felt that removal of the pleural fluid would improve their performance status to 1 or better. MPE is defined as either histocytologically proven pleural malignancy or an exudative effusion with no other cause in a patient with known primary extra-pleural malignancy.

### Exclusion criteria

Exclusion criteria includes age < 18 years; unfit to undergo surgical procedure (American Society of Anaesthesiology Score ≥ 4); significant loculations likely to preclude effective drainage via IPC; pleural infection; chylothorax; pregnancy or lactation; uncorrectable bleeding diathesis; and previous ipsilateral lobectomy/pneumonectomy and inability to consent or comply with the protocol.

### Informed consent

Potential participants will be provided with verbal information and the participant information and consent form (PICF) to read by a member of the research team. They will be given time to ask questions and to discuss the study with family/carers and their GP.

### Interventions

#### VATS arm

Participants randomised to the VATS arm will undergo surgery within two weeks of randomisation. VATS is usually performed in an operating theatre, using either general anaesthesia or sedation. The pleural fluid will be removed, and adhesions can be divided. Assessment of lung re-expansion will be performed intra-operatively. If lung re-expansion is adequate (as judged by the operating surgeon), a variety of techniques may be employed to induce pleurodesis, including, but not limited to, talc poudrage and mechanical abrasion. Decortication may be performed if deemed appropriate and feasible. A chest drain (non-IPC) will be left in situ after the surgery. Post-operative care will be performed as per local practice.

#### IPC arm

IPCs will be inserted as per local protocols. If full lung expansion (see Trapped Lung SOP, Additional file 1) is achieved after drainage of the effusion (typically assessed 1 day post-insertion), sterile talc (4–5 g, graded) will be instilled as a slurry via the IPC, and subsequent management (e.g., use of suction) will follow local protocols for talc slurry pleurodesis at each site. Daily drainage with suction bottles will then be performed for 7 to 10 days after talc instillation until outpatient review. Successful development of pleurodesis is defined as fluid output of less than 50 ml on three consecutive drainage attempts via IPC without evidence of fluid accumulation on imaging. The IPC can be removed when pleurodesis is achieved. In the case of ongoing pleural fluid production and drainage via the IPC, daily drainage can continue or be adjusted to a symptom-guided drainage regimen as determined by the treating team.

If the lung does not fully expand following complete evacuation of pleural fluid (see Trapped Lung SOP, Additional file 1), the participant will be discharged on a daily drainage regimen to allow the best chance of full lung re-expansion, which will be assessed at outpatient follow-up. In the event of full lung expansion (≥ 75% pleural apposition) in association with ongoing fluid production, talc instillation via IPC can be considered (as described above) and followed by daily drainage and review at 7–10 days after the attempted pleurodesis. If the lung remains trapped, further drainage will follow a symptom-guided drainage regimen. At any time, if the output is < 50 ml/drainage on three consecutive drainage attempts at least 24 hapart, with no evidence of symptomatic fluid accumulation, the IPC can be removed.

### Standard care and follow-up

Participants in both arms will be managed by their own clinical teams and receive all other usual medical treatment (including chemotherapy and radiotherapy). All participants and carers will have access to the research team should any concerns arise. This cohort will remain under the continued care of a physician or thoracic surgeon following trial completion.

Participants are advised prior to enrolment that they are free to withdraw from the trial at any time if they so wish. Following randomisation, changes to the intervention plan may be made at the discretion of the treating physician or due to participant request.

### Strategies to improve adherence to interventions and participant retention

Potential participants, as part of the informed consent process, will have the study procedures and visit plan discussed in detail. An emphasis will be placed on the need for attendance at all study visits. Where participants do not attend planned study visits, the research team will make contact and book an unscheduled visit if required.

### Outcomes

Data will be collected by the site investigators from the participant at follow-up visits (see 16) and from their hospital record.

#### Primary endpoint

The primary outcome is the need for an ipsilateral pleural intervention within 12 months of randomisation for symptomatic recurrence of pleural effusion. Pleural intervention will be defined as an ipsilateral surgical procedure, chest drain insertion or thoracentesis with therapeutic intent.

#### Secondary endpoints

- Repeat ipsilateral pleural intervention including diagnostic aspiration, e.g., for the investigation of possible infection will be identified as above
- Time to symptomatic effusion recurrence will be measured from the date of procedure, by performing radiological investigations if the participant describes worsening dyspnoea at a study visit. Recurrent effusion is defined as greater than 25% increase in opacification on chest x-ray (CXR) on the side of the intervention, and evidence of pleural fluid on ultrasound
- All-cause and pleural-related hospital days will be measured. Length of stay post-procedure and hospitalisation for any cause (except for elective admissions for chemotherapy) will be recorded for all participants post-enrolment until the end of follow-up period or death. Admissions will be analysed as total admission days (and number of episodes) and as pleural-related admission days
- Degree of breathlessness will be measured using a 100-mm visual analogue scale (VAS), which is a validated score of dyspnoea in MPE [23]. The VAS is a 100 mm line anchored with “no breathlessness” at 0 mm and “worst breathlessness imaginable” at 100 mm and will be done during the aforementioned outpatient reviews. Two independent researchers will measure all VAS scores and the mean score will be calculated. Differences in scores of more than 3 mm in both measurements will be repeated by the same observers
- Pain will be assessed using a 100-mm VAS with timing and measurement as for breathlessness
- Quality of Life (QoL) will be measured using two instruments, the EQ-5D-5L questionnaire and VAS QoL, both employed as primary or secondary end-points in previous RCTs [23, 40]. VAS QoL records self-rated quality of life on a 100 mm line, anchored with “best imaginable quality of life” at one end-point and “worst imaginable quality of life” at the other. QoL will be measured at each study visit
- Physical activity patterns will be evaluated by a well-validated triaxial accelerometer (ActiGraph GT3X+, Pensacola, FL, USA) providing an indication of functional status [41]. Standard data processing will be applied and the weekly duration of low, moderate, and vigorous-intensity physical activity determined in accordance with established ranges. This will be performed at the lead-site only
- Adverse events will be recorded from time of enrolment until end of follow-up or death. An adverse event is defined as any complication associated with the IPC or VATS pleurodesis, including, but not limited to, pleural infection, cellulitis, pain, symptomatic loculation +/− requirement of intrapleural fibrinolytics, tube blockage, catheter tract metastases, parenchymal air leak, etc., and any peri/post-procedural complications such as prolonged air-leak, atelectasis, pneumonia, cardiovascular complications, acute kidney injury, or drop in haemoglobin requiring transfusion
- Economic analysis will be performed by obtaining inpatient costs from the hospital. Outpatient pleural care costs and equipment utilisation will be noted (Western Australia only)
- Overall survival will be recorded from date of enrolment to death or end of study follow-up

### Participant timeline

Baseline data will be collected once informed consent has been obtained. Data collected will include demographics, ASA, ECOG performance status, questionnaires (VAS breathlessness, VAS QoL, VAS pain, EQ-5D-5L), comorbidities, malignancy type, stage and treatment, baseline bloods (full blood picture, albumin, lactate dehydrogenase, C-reactive protein), baseline CXR findings, baseline ultrasound thorax and pleural effusion data including laboratory results and previous interventions.

**Fig. 1:**
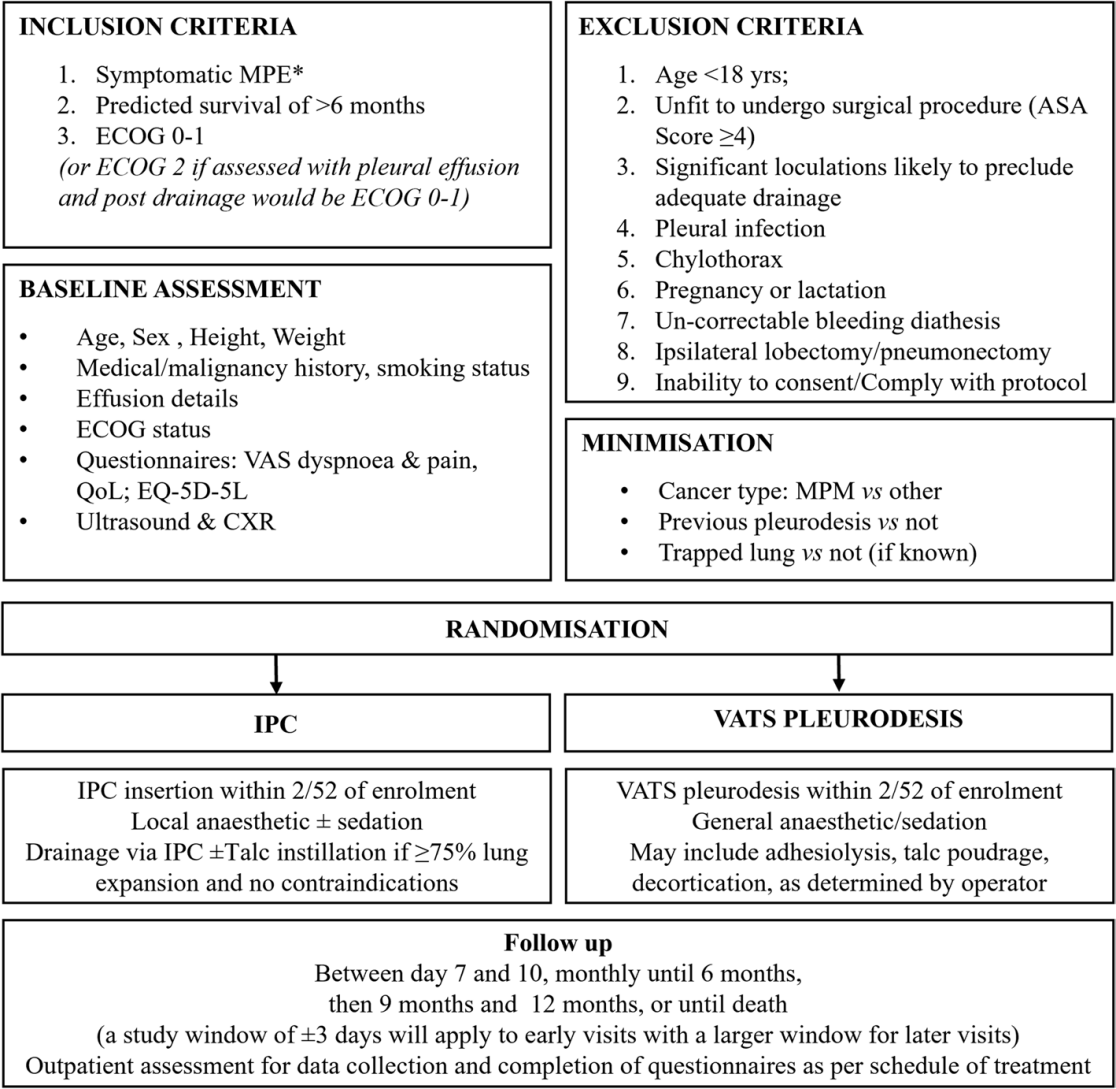
Study flow chart. MPE, malignant pleural effusion

The assigned intervention will be scheduled to occur within 2 weeks of randomisation. Participants will be followed up between day 7 and 10 post-procedure, monthly to 6 months, at 9 months, and at 12 months or death if sooner (Table 1, Fig. 1). There will be a visit ‘window’ initially of ±3 days surrounding the early visits. Later visit dates will have a larger visit window.

**Table 1:** Schedule of treatment for each visit and follow-up procedures

	Study period								
	Pre-procedure		Post-procedure						
	Enrolment	Within 2/52	Days post-procedure				Months		
Timepoint	***T-1***	***T0***	***D1***	***Discharge***	***D7-10***	***D28***	***2–6***	***9***	***12***
**Enrolment:**									
**Clinical assessment**	X	X	X		X	X	X	X	X
**Bedside ultrasound**^**a**^	X				X	X	X	X	X
**Informed consent**	X								
***Baseline data collection***	X								
***Blood tests***^**b**^	X								
***CXR***^**c**^	X				X	X	X	X	X
**Randomisation (IVRS)**	X								
**Interventions:**									
***Drainage procedure (VATS or IPC)***		X							
**Assessments:**									
***Questionnaires (VAS QoL, dyspnoea, pain; EQ5D5L)***	X				X				X
***Logbook commencement***			X						
***Logbook collection***						X			
***Procedure-related data collection***				X					
***ActiGraph to participant—lead site only***			X			X	X		
***Assessment for pleurodesis***^**d**^					X	X	X	X	X
***Assessment for re-intervention***			X		X	X	X	X	X
***Adverse event review***			X		X	X	X	X	X

^a^Bedside ultrasound mandatory at baseline only

^b^Blood tests to include FBC, albumin, LDH, and CRP

^c^CXR mandatory at baseline, days 7–10, and months 1, 3, 6, 9, and 12

^d^IPC arm only. Spontaneous pleurodesis is defined as < 50 ml drain output on 3 consecutive drainages at least 24 h apart

### Sample size

This study will enrol 158 patients to detect a 5% reintervention rate in optimised IPC vs 25% reintervention rate in VATS (based on previous studies [13, 22, 23]). The sample size calculation was carried out using an anticipated Fishers exact test to compare these proportions in each of the two groups. With a 5% significance level and a power of approximately 90%, we would need 75 patients per group with an additional 4 patients per group to allow for dropouts (5% anticipated) giving a total of 158 patients. Withdrawal or loss to follow-up only occurred in 4.8% (7/146) patients in our previous AMPLE-1 trial of MPE [22].

### Randomisation and blinding

Participants will be randomly assigned (1:1) to either IPC (with talc if suitable) or VATS pleurodesis. Randomisation will include minimisation for (i) cancer type (mesothelioma vs non-mesothelioma); (ii) previous pleurodesis (*vs* not); and (iii) trapped lung, if known (*vs* not known). The nature of the intervention means that investigators and patients cannot be blinded to the treatment arms. The National Health and Medical Research Council Clinical Trials Centre, Sydney, Australia, provides the randomisation setup via their automated telephone-based interactive voice response services. Where possible, data analysts will be blinded for analysis of the outcomes although the very different profiles of treatment outcomes of the allocated treatments will make complete blinding difficult.

### Data management and safety

Data will be entered into a secure database and a system of data validation checks will be implemented and applied. The accuracy of the data will be verified through data monitoring and comparison to source documents. Data entered will be checked by a second staff to ensure accuracy.

All physical documentation will be stored in a secure environment in line with the Australian Code for the Responsible Conduct of Research for clinical trials and local policy guidelines for research data archiving. All procedures for the handling and analysis of data will be conducted using GCP ICH guidelines, the National Statement on Ethical Conduct in Human Research (2007)—*Updated May 2015*, and local policies and procedures for the handling and analysis of data.

### Statistical plan

Data will be analysed on an intention-to-treat basis and per protocol basis. The primary outcome will be analysed using a Fisher’s exact test for comparing two proportions and subsequent logistic regression analyses allowing adjustments for minimisation variables. A secondary analysis of the primary outcome will use a competing risks time to event analysis with the competing risk of death. For the secondary outcomes, e.g., hospital days and VAS measurements, the difference between the groups will initially be examined using two sample *t*-tests and further adjustments will be made for minimisation variables. Subsequent analyses will consider the repeated VAS scores measured on the same patient and missing VAS scores (assuming that missing scores are missing at random) and will include a time by treatment interaction term along with random intercepts and time effects as appropriate. An interim analysis is planned after the recruitment and follow-up of 60–80 participants.

The research team will document as accurately as possible the reasons for any non-completion or missing data, thereby minimising truly absent data. The expected dropout rate from patient death has been factored into the power calculation and is based on survival figures and previous studies.

### Ethics

The trial has been approved by an ethics committee for all sites (see declarations). The investigators will receive approval from the ethics committee for any amendment to the protocol and ensure it is signed by any patient subsequently entering the trial and those currently in the study, if affected by the amendment.

### Trial monitoring and oversight

The trial steering committee (TSC) will be responsible for the supervision of the trial in all its aspects, including completion of the trial to clinical and ethical standards. Members of the TSC include the principal investigator, selected investigators from each site, external independent members, a consumer representative, and the trial coordinator.

The data safety and monitoring board (DSMB) will ensure the safety of study participants through monitoring of ethical conduct of the study and adverse events and consider new data (recently published studies) that may determine the validity of study continuation. The DSMB includes an independent chairperson and other independent members, one of whom is a statistician.

### Sponsorship

The study is sponsored by the Institute for Respiratory Research a not-for-profit organisation.

Contact details: Mr Bi Lam, Finance Manager, Level 2, 6 Verdun Street, Nedlands WA 6009

t| +61 8 6151 0877 e| bi.lam@resphealth.uwa.edu.au

The study investigators/institutions will permit trial-related monitoring, audits, and regulatory inspections, providing direct access to source data/documents. This may include, but is not limited to, review by external sponsors, Human Research Ethics Committees, and institutional governance review bodies.

### Adverse event reporting and harms

Although both arms of this study involve procedures that are standard of care, participants will be carefully monitored for the development of adverse events, which will be documented according to standard “Adverse Event Reporting”. Any serious adverse events, whether related to the intervention or not, will be reported, documented, and reported to the DSMB. Adverse events will be followed up until resolution or documented as not resolved on the adverse event log at study completion.

All deaths, anticipated or unanticipated, will be discussed with the DSMB. The committee determines whether significant benefits or risks have been uncovered which may have an impact on the feasibility and/or ethical conduct of the study. The DSMB will also help to ensure the scientific integrity of the study by reviewing the quality of the data it uses to make its decisions.

### Plans for dissemination

It is expected that the study results will be published in a peer-reviewed journal. Presentations at national and international conferences are anticipated.

## Discussion

IPC and VATS are both well-established treatments for MPE, but the choice of procedure is often dictated by clinician bias and available resources. The results of this study will provide the first objective evidence-based guidance on which treatment would be the best for patients who are suitable for both interventions.

## Trial status

Protocol version: Version 3.00/4.02.19

Date recruitment began: 23 April 2019

Estimated recruitment completion date: 31 December 2024

## Supplementary Information

**Additional file 1.** Standard Operating Procedure AMPLE-3 Trial: Diagnosing Trapped Lung.

## Data Availability

The research team at the lead site will have access to the final trial dataset. Supporting data including standard operating procedures, details of data management procedures, case report forms, and datasets generated and/or analysed during the current study will be available to the scientific community with as few restrictions as possible, while retaining exclusive use until publication of major outcomes. Data requests from qualified researchers should be made to YCGL (gary.lee@uwa.edu.au).

## Abbreviations

ASA: American Society of Anaesthesiologists score
CXR: Chest x-ray
DSMB: Data and Safety Monitoring Board
ECOG: Eastern Cooperative Oncology Group
IPC: Indwelling pleural catheter
MPE: Malignant pleural effusions
QoL: Quality of life
SAE: Serious adverse event
TSC: Trial Steering Committee
VAS: Visual analogue scale
VATS: Video-assisted thoracoscopic surgery
