# Supplementary material for: Australasian Malignant PLeural Effusion (AMPLE)-3 trial: study protocol for a multi-centre randomised study comparing indwelling pleural catheter (±talc pleurodesis) versus video-assisted thoracoscopic surgery for management of malignant pleural effusion
Source: Trials. 2022 Jun 27;23:530. doi: 10.1186/s13063-022-06405-7 (PMC9235203; doi:10.1186/s13063-022-06405-7)
Supplement: Supplementary file 1 — Additional file 1. Standard Operating Procedure AMPLE-3 Trial: Diagnosing Trapped Lung. [file 13063_2022_6405_MOESM1_ESM.pdf]

Standard Operating Procedure AMPLE-3 Trial:
Diagnosing Trapped Lung

Trapped lung diagnosis required in the study in relation to,

1. Study entry minimisation criteria - if known.
2. Confirmation of absence of trapped lung prior to talc instillation in IPC arm 6-48 hrs post-IPC insertion.

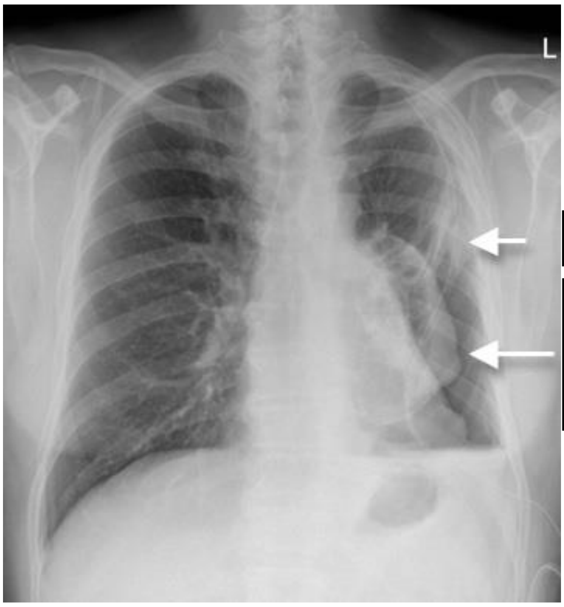

IPC in situ

Air within the pleural space occupying more than 25% of hemithorax post-drainage

Image: Example of Trapped Lung

DIAGNOSIS PATHWAY

IPC inserted and drained to dryness

Confirm complete drainage of pleural space on x-ray

Air/fluid in the pleural space occupying at least 25% of hemithorax and no evidence of air leak

YES → **Trapped lung**

NO → **No Trapped lung**
